# Pharmacokinetics of mefloquine administered with artesunate in patients with uncomplicated falciparum malaria from the Brazilian Amazon basin

**DOI:** 10.1186/s12936-018-2416-0

**Published:** 2018-07-16

**Authors:** Michelle V. D. Ferreira, José L. F. Vieira, Eduardo D. Almeida, Juan G. B. Rivera, Margarete S. M. Gomes, André M. de Siqueira

**Affiliations:** 1 Pharmacy Faculty, Pará Federal University, Augusto Correa Street 01, Campus Universitário do Guamá, Belém, Pará Brazil; 2 Laboratory of Public Health of Macapa, Av. Adilson José Pinto Pereira, 907 Macapá, Amapá Brazil; 3 Fiocruz, Av. Brasil, Manguinhos, Rio De Janeiro, Brazil

**Keywords:** Malaria, Mefloquine, Pharmacokinetics

## Abstract

**Background:**

A fixed-dose combination of mefloquine with artesunate was evaluated in cases of falciparum malaria in the Brazilian Amazon basin with acceptable efficacy, safety and tolerability. However, there are no data on the pharmacokinetics of mefloquine in this coformulation in Brazil, which is valuable to evaluate whether *Plasmodium* is exposed to an effective concentration of the drug.

**Methods:**

A prospective, single-arm study was conducted in male patients with slide-confirmed infection by *Plasmodium falciparum* using two tablets of a fixed-dose combination of artesunate (100 mg) and mefloquine base (200 mg) once daily and over 3 consecutive days. Serial blood samples were collected at admission and throughout 672 h post-administration of the drugs. Mefloquine was measured in each blood sample by high-performance liquid chromatography. The pharmacokinetic parameters were determined by non-compartmental analysis.

**Results:**

A total of 61 patients were enrolled in the study and 450 whole blood samples were collected for mefloquine measurement. The mefloquine half-life was 10.25 days, the maximum concentration (C_max_) was 2.53 µg/ml, the area-under-the-curve (AUC_0–∞_) was 359 µg^/^ml h, the observed clearance (Cl/f) was 0.045 l/kg/h and the volume of distribution (V/f) was 14.6 l/kg. Mefloquine concentrations above 0.5 µg/ml were sustained for a mean time of 9.2 days.

**Conclusion:**

The pharmacokinetic parameters of mefloquine determined in the study suggest an adequate exposure of parasite to mefloquine in the multiple oral dose regimen of the fixed dose combination of mefloquine and artesunate.

## Background

Malaria remains one of the major public health issues worldwide. In Brazil, most of the cases occur in the Amazon basin, with about 148,000 cases reported each year. *Plasmodium falciparum* is responsible for 15% of the burden of the disease in this endemic scenario, with approximately 13,000 cases each year [1]. The treatment is based on artemisinin-based combination therapy (ACT). In Brazil, artemether with lumefantrine (Coartem^®^) is recommended by the local health authorities as a first-line therapy for uncomplicated falciparum malaria in a 3-day course of 80 and 480 mg every 12 h in patients weighing 35 kg or more [2]. To date there have been no signs of loss of Coartem^®^ efficacy in the Brazilian Amazon basin, however, ongoing vigilance is needed to detect the emergence of resistance to artemisinin or partner drugs.

Recent studies done with a fixed-dose formulation of artesunate with mefloquine (MQAS) manufactured by Farmanguinhos (Fiocruz, Brazil) in single 3-day doses of two tablets of 100 mg of artesunate and 200 mg of mefloquine base have shown acceptable efficacy, safety and tolerability of MQAS in uncomplicated falciparum malaria cases. Moreover, the coformulation reduces the pill number compared to the standard treatment. Thus, MQAS may be a potential alternative to Coartem^®^ in this endemic setting [3, 4].

Mefloquine, the partner drug, is a quinolone methanol compound effective against the asexual blood stage of *P. falciparum* introduced in 1984 for clinical use in Thailand [5]. The pharmacokinetic (PK) parameters of the drug were determined in several population groups under different exposure conditions. The comparisons among studies revealed significant variations of PK parameters, which are due to the oral bioavailability of the drug related to the commercial formulation, malabsorption, vomiting and dose regimens. Also, the co-administration of artesunate with mefloquine modifies the volume of distribution, the maximum concentration and the clearance rate of mefloquine. These changes in PK parameters may be clinically relevant since they can modify the exposure of the parasite to the adequate concentrations of anti-malarial drugs in the blood and, consequently, may impair the therapeutic response [6–14]. The minimum concentration required to cure uncomplicated falciparum malaria remains uncertain, but blood concentrations above 0.5 μg/ml are associated with high rates of cure [6].

As the clinical studies of efficacy and tolerability of a new anti-malarial drug formulation should be accompanied by the estimation of exposure of parasite to adequate concentrations of the anti-malarial drug, it was investigated in the present study the pharmacokinetics parameters of mefloquine given as a fixed-dose formulation with artesunate once daily in patients with uncomplicated falciparum malaria from the Brazilian Amazon basin.

## Methods

### Study site and subject

This was an open-label, single arm study carried out at Reference Centre for Tropical Diseases in the municipality of Macapa, AP, in the Brazilian Amazon Basin. Patients recruited for attendance in the health facility came from rural communities or gold mines located at the border between Brazil and French Guyana. Inclusion criteria for enrolment in the study were: adult male > 18 years of age with slide-confirmed mono-infection by *P. falciparum*. The exclusion criteria included patients with signs or symptoms of severe disease (jaundice, renal impairment, severe anaemia, altered level of consciousness), parasitaemia over 5%, mixed infections, history of psychiatric disorders, overweight and underweight, altered levels of creatinine, altered levels of aspartate aminotransferase (AST) or alanine aminotransferase (ALT), known hypersensitivity or allergy to mefloquine or artesunate and use of mefloquine in the previous 90 days.

### Treatment and follow-up

Each patient received two tablets of a fixed-dose combination of mefloquine with artesunate (Farmanguinhos, Brazilian Health Office) containing 100 mg of artesunate and 200 mg of mefloquine base per tablet, once a day and over 3 consecutive days [2]. In all days of treatment, the administration of ASMQ was supervised by the research team, as well as, for vomiting, diarrhoea and other adverse reactions that were carefully monitored within 2 h after ASMQ intake. Patients were requested to return for blood sampling and clinical evaluation on days 1, 3, 5, 7, 14, 21, 28, and 42 after inclusion in the study.

### Blood sample collection

Serial venous blood samples (4 ml) were taken from each patient for mefloquine measurement before the commencement of the treatment (D0) and on days 1, 3, 5, 7, 14, 21, 28, and 35. On day 1, a blood sample was collected from each patient 30 min before anti-malarial drug intake. A similar time of blood sampling was adopted for all days of the study for all patients. After collection, whole blood samples were immediately stored at -80 C until analysis.

### Measurement of mefloquine

Mefloquine was measured by a reversed-phase HPLC system with an ultraviolet detector (Pro Star—Varian, Walnut, CA, USA) after liquid–liquid extraction from the whole blood with methyl tert-butyl ether at pH 4.0. The separation was carried out on a reversed-phase column (ODS C18 4.6 × 250 mm id 5 µm; Supelco Inc. Bellefonte PA, USA). The mobile phase consisted of acetonitrile–phosphate buffer (0.1 ml/l; pH 2.5) (42:58). Quinidine (2.5 μg/ml) was used as internal standard. Whole blood spiked with mefloquine in concentrations of 0.05, 0.1, 0.5, 1, 2, and 4 μg/ml were used to estimate the within-day and day-to-day coefficients of variation as well as the recovery of the method. The limit of detection was considered as the lowest concentration of mefloquine that may be differentiated from the background noise of the ultraviolet detector and the limit of quantification was considered as the lowest concentration of mefloquine that was determined with a coefficient of variation below 10%. The assay was linear from 0.05 to 4 μg/ml. The within-day and day-to-day coefficients of variation were 6.7 and 8.1%, respectively. The limit of quantification was 0.05 μg/ml and the limit of detection was 0.03 μg/ml. The mean recovery was 90%. The stability of blank whole blood spiked with mefloquine (0.1 μg/ml) was 60 days at − 80 °C. There was no significant interference of primaquine, quinine, chloroquine, desethyl-chloroquine, carboxy-mefloquine, and acetaminophen in the detection of mefloquine [15].

### Evaluation of efficacy

Parasite count was done in Giemsa-stained thick films every day until it became negative and then on days 7, 14, 21, 28, 35, and 42. An experienced microscopist examined the blood films using 100× (oil immersion) objectives. The parasite density was expressed as the number of parasites per μl of blood, which was derived from the number of parasites per 200 white blood cells, considering a total white blood cell count of 8000. The limit of detection of the parasites was 40/µL [16]. Clinical and parasitological outcomes were based on the standardized WHO protocols [17].

### Data analysis

The data were analysed by non-parametric methods. A non-compartmental pharmacokinetic analysis was used to calculate the pharmacokinetics parameters, which were estimated separately for each patient enrolled in the study. The maximum concentration (Cmax) was obtained directly from the whole blood concentration–time profile. The AUC_0−t_ was estimated from the time of drug administration to the time of the last measurable concentration by using the linear trapezoidal rule and the extrapolation to infinity AUC_0−∞_ was determined by dividing the last measurable mefloquine concentration by λZ. To obtain the terminal elimination rate constant (λZ), the mefloquine concentrations were log transformed and fitted a linear regression model to the terminal phase of the concentration–time profiles by using the method of least squares. The terminal elimination half-life (t_1/2_) was estimated by dividing ln2 by λZ. The apparent oral clearance per fraction of drug absorbed (CL/f) was derived from the ratio of the dose to AUC_0−∞_. The apparent volume of distribution (V/f) was estimated from CL/f divided by λZ. Data were analysed using WinNonlin (version 3.3; Pharsight Corp, Mountain View, CA, USA).

### Ethical statement

The study was approved by SEAMA Faculty Ethical Committee under the number 079/08. All patients enrolled in the study were informed about the goals as well as the risks and benefits of the study. All patients provided written informed consent before entering the study following national guidelines.

## Results

A total of 61 patients were recruited for the study and 495 whole blood samples were collected and analysed for mefloquine with an average of 8 time points (range 5–9 time points) for each patient. A total of 54 samples were missing due to loss to follow-up of participants.

The median total dose of mefloquine received by patients of 21.05 mg/kg (range 15.3–24 mg/kg) was sufficient to achieve curative concentrations of the drug. The geometric mean of parasites at admission was 1900 µl (range 800–5100 µl). Parasitaemia was cleared rapidly in all patients and there was no recrudescence by day 42, suggesting a high therapeutic efficacy of MQAS to treat uncomplicated falciparum cases in this endemic area. Furthermore, there were no reports of vomiting or diarrhoea, but 7 patients (17.5%) showed nausea or insomnia during the treatment. The baseline characteristics of participants are shown in Table 1.

**Table 1:** Baseline characteristics of patients

Characteristics	Patient (n = 61)
Age (years)	31 (22–41)
Weight (Kg)	57 (50–78)
Parasitaemia at admission (µl) geometric mean	1900 (800–5100)
Time for fever clearance (h)	18 (12–28)
Time for parasite clearance (h)	24 (12–60)
Reappearance of parasite on day 42 (%)	0
White blood cells count (µl)	7400 (5200–12,700)
Erythrocyte count × 10^6^ (ul)	4.23 (3.9–4.9)
Hemoglobin (g/dl)	11.3 (10–14)
Creatinine (mg/dl)	0.7 (0.5–0.9)
AST U/l	35 (15–40)
ALT U/l	46 (12–47)

Results are expressed as median and ranges

**Fig. 1:**
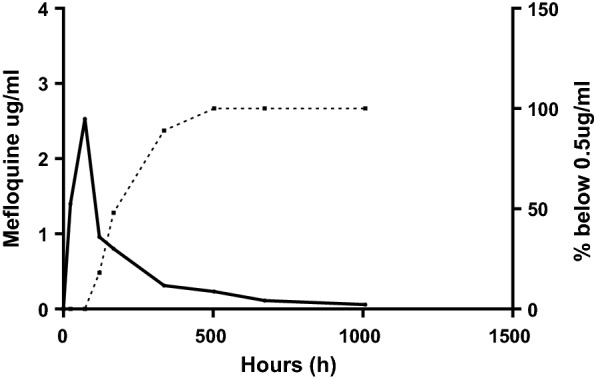
Concentration-time profile of mefloquine (left axis) and the percentage of whole blood samples with mefloquine concentrations below 0.5 µg/ml (right axis)

All pre-dose samples (d0) had undetectable levels of mefloquine. The median concentrations of mefloquine were 1.4 μg/ml (range 0.6–3.4 μg/ml), 2.53 μg/ml (range 1.5–3.88 μg/ml), 0.96 μg/ml (range 0.54–2.18 μg/ml), 0.8 μg/ml (range 0.5–1.4 μg/ml), 0.31 μg/ml (range 0.1–1.14 μg/ml), 0.23 μg/ml (range 0.06–0.6 μg/ml), 0.11 μg/ml (range 0.06–0.27 μg/ml) and 0.067 μg/ml (range 0.05–0.07 μg/ml) on days 1, 3, 5, 7, 14, 21, 28, and 42, respectively. Mefloquine concentrations above 0.5 μg/ml were sustained for a mean time of 9.2 days. The concentration–time profile of mefloquine is shown in Fig. 1. The pharmacokinetic parameters of mefloquine determined by non-compartmental analysis are shown in (Table 2).

**Table 2:** Pharmacokinetic parameters of mefloquine in the presence of artesunate

Parameter	Value
C_max_ (µg/ml)	3.1 (1.5–3.8)
AUC_0−t_ (µg/ml h)	480 (330–60)
AUC_0−∞_ (µg/ml h)	484 (363–777)
Cl/*f* (liters/kg/h)	0.04 (0026–0058)
*V/f* (liters/kg)	13.2 (6–19)
t_1/2_ (days)	10.1 (7–16)

Results are expressed as median and range

## Discussion

The emergence and dissemination of ACT resistance in Southeast Asia constitute a threat to reduce the burden of falciparum malaria worldwide. The search for new compounds with anti-malarial action and the development of new ACT formulations has been a plausible strategy, adopted by several countries, to the fight against the disease. In this line, a fixed-dose formulation of MQAS was developed by Farmanguinhos, Brazil. In the current study, the pharmacokinetic parameters of MQAS were estimated. Furthermore, this is the first study to use a large data set to demonstrate the pharmacokinetic parameters of mefloquine in Brazilian patients with uncomplicated falciparum malaria. All patients had low parasite density and mild signs and symptoms of the disease, which is in line with the pattern of uncomplicated cases in the Brazilian Amazon Basin [18–20].

In the study, the mean time for parasite clearance is aligned to studies in areas where MQAS remains effective. Moreover, there was no recrudescence of the infection by day 42. These results suggest a high therapeutic efficacy of the fixed-formulation of MQAS in this endemic area [5, 8, 9, 21, 22]. However, there is a broad range of the total dose of mefloquine per body weight in these patients, which can lead to underdosing, contributing to the emergence of resistance. Therefore, it is recommended that there be an adjustment of the dosing regimen by the weight of patients with this fixed-dose formulation of MQAS.

The split-dose regimen of mefloquine was well tolerated by the patients with a low occurrence of side effects [8, 23–25]. In fact, patients reported only nausea and insomnia whereas vomiting was not reported before 2 h after anti-malarial drugs intake.

The non-compartmental modelling of mefloquine concentration–time profile was used in the present study. This approach had been applied with success in studies of the pharmacokinetics of mefloquine [7–9, 25]. The means values of the Cmax and the AUC0 → ∞ suggested a good oral absorption of mefloquine in this split-dose regimen. The Cmax of 3.1 µg/ml is within the range of values reported in uncomplicated falciparum malaria cases under similar dose regimen [8]. Furthermore, after the normalization of the dose by the weight of patients, the AUC0 → ∞ agrees with studies in patients with uncomplicated falciparum malaria [8, 25, 26].

The coformulation MQAS involves a rapidly eliminated and fast-action artemisinin derivative and a slowly eliminated drug that kills the remaining parasites [27]. Mefloquine has a low systemic clearance, a large volume of distribution, and a long terminal half-life [21]. In the present study, the volume of distribution was 13.2 l/kg, the systemic clearance was 0.04 l/kg/h and the terminal half-life was 10.1 days. These characteristics allow the exposure of *P. falciparum* for a long period to effective concentrations of the drug in the blood.

The comparisons of pharmacokinetic parameters of the present study with those derived from African and Thai patients presented some similarities. For instance, the mean half-life was comparable to mean half-life of 9.4 days found in Thai patients [8, 12]. The systemic clearance was also comparable to reports of Thai patients [12]. Finally, the volume of distribution was high when compared to Thai patients [25, 28]. The potential difference in the disposition of mefloquine between healthy individuals and patients with falciparum malaria was also found in the present study, as the terminal half-life of mefloquine was considerably lower than in healthy Thai and African volunteers [12, 29].

Furthermore, a considerable intra-individual and inter-individual variation of pharmacokinetic parameters of mefloquine was found in the study. A plausible explanation is that the data were normalized only by the weight of patients and not by other confounding factors, including age, parasitaemia at admission, time of fever clearance, and body mass index. This was considered the main limitation of the current study [8, 9, 30, 31].

## Conclusion

The pharmacokinetic parameters of the coformulation of artesunate with mefloquine in a fixed-dose formulation suggest an adequate exposure of *P. falciparum* to the fixed dose, which was corroborated by the efficacy of the treatment. Thus, artesunate-mefloquine is a reliable alternative treatment to Coartem^®^ in this endemic area.
